# Y chromosome microdeletions in infertile men with idiopathic oligo- or azoospermia

**DOI:** 10.1186/1743-1050-3-1

**Published:** 2006-01-30

**Authors:** Ali Hellani, Saad Al-Hassan, Muhammed A Iqbal, Serdar Coskun

**Affiliations:** 1 Department of Pathology and Laboratory Medicine, King Faisal Specialist Hospital and Research Center, Riyadh, Saudi Arabia; 2 Department of Obstetrics and Gynecology, King Faisal Specialist Hospital and Research Center, Riyadh, Saudi Arabia

## Abstract

About 30–40% of male infertility is due to unknown reasons. Genetic contributions to the disruption of spermatogenesis are suggested and amongst the genetic factors studied, Y chromosome microdeletions represent the most common one. Screening for microdeletions in AZFa, b and c region of Y chromosome showed a big variation among different studies. The purpose of this study was to investigate the prevalence of such deletions in Saudi men. A total of 257 patients with idiopathic oligo- or azoospermia were screened for Y chromosome microdeletions by 19 markers in AZF region. Ten (3.9%) patients had chromosomal rearrangements, six of them showed sex chromosome abnormalities and four patients had apparently balanced autosomal rearrengements. Eight of the remaining 247 patients (3.2%) with a normal karyotype and no known causes of impaired spermatogenesis had Y chromosome microdeletions. Among these, six patients had deletions in AZFc region, one case had a deletion in AZFb and another had both AZFa and AZFc deletions.

In conclusion, our study shows that Y chromosome microdeletions are low in our population. We also report for the first time a case with unique point deletions of AZFa and AZFc regions. The lower frequency of deletions in our study suggest that other genetic, epigenetic, nutritional and local factors may be responsible for idiopathic oligo- or azoospermia in the Saudi population.

## Introduction

Approximately 10–15% of couples are affected by infertility. A male factor can be diagnosed in approximately 50% of them and about 30–40% of male infertility is due to unknown origin [1]. Little is known about the genetic disorders that cause disruption of spermatogenesis. Early cytogenetic studies showed that microscopic deletions in the long arm of Y chromosome are responsible for azoospermia [2]. With the advancement in molecular biology, three non-overlapping regions named "azoospermia factors" (AZFa, b, c from proximal to distal Yq region) have been defined as spermatogenesis loci [3]. It is now widely accepted that deletions within those three regions severely diminish the sperm production [4]. The power of polymerase chain reaction (PCR) and the availability of sequence-tagged site (STS) maps made possible the detection of interstitial deletions in Yq11 region that was invisible by karyotyping [5]. Such microdeletions have been reported with varied prevalence in different populations and studies [6].

The introduction of intracytoplasmic sperm injection (ICSI) into the treatment of male factor infertility [7] permitted the use of sperm from oligo- or azoospermic patients to achieve successful fertilization and pregnancies. This technology raised both hopes for infertile men to have their own child, which would be otherwise impossible and concerns to medical professionals about inheritance of Y chromosome deletions and genetic disorders to the next generations. Although these patients have a right to father a child with the current technology, however, they should be counseled about the risk they are transmitting to their offspring [8]. The aim of this study was to screen for Y chromosome microdeletions in infertile males who were candidate for ICSI treatment and with idiopathic oligo- or azoospermia.

## Materials and methods

### Patients

A total of 257 Saudi infertile patients with idiopathic oligozoospermia or azoospermia were enrolled in the study. Patients were recruited from the infertility clinic in King Faisal Specialist Hospital and Research Center. All males were subjected to complete physical exam, semen analysis and endocrine work-up (FSH, LH, TSH, testosterone, prolactin). Patients with two consecutive specimens with sperm count less than 10 × 10^6^/ml were marked as candidate for the study and referred to an andrologist. These candidates were checked for the history of relevant medical disorders e.g. diabetes, sickle cell disease, liver and renal disease, radiation, endocrine abnormality (e.g. prolactinoma, hypogonadotropic hypogonadism); exposure to toxins and/or medication affecting spermatogenesis; gross dismorphic abnormalities; acquired and congenital structural defects of urogenital system (cystic fibrosis, Young syndrome); history of surgical intervention of genital tract obstruction/dysfunction. Patients with positive evidence of any of the above conditions were excluded from the study. Upon diagnosing idiopathic oligo- or azoospermia, patients were counseled for the study and asked to sign an informed consent form for karyotyping and Y chromosome microdeletion analysis. DNA isolated from 53 Saudi men with normal semen analysis was used as controls. An IRB approval was obtained for the study.

### Karyotyping

Cytogenetic analysis was performed from phyto-haemagglutinin stimulated lymphocyte cultures by routine laboratory protocol. For microscopic analysis, metaphase chromosomes were stained with trypsin-Giemsa technique [9]. For chromosome analysis, 10 to 20 cells were analyzed; and two to five metaphases were karyotyped. For the definition of chromosomal abnormalities, International System for Human Cytogenetic Nomenclature (ISCN) was followed [10].

### DNA extraction and PCR

Genomic DNA was prepared from peripheral blood samples using DNAzol according to the manufacturer instructions (InVitrogen, USA). A total of 19 specific targeted sequences (STSs-3 markers in AZFa, 7 markers in AZFb and 9 markers in AZFc) have been assessed using polymerase chain reaction (PCR) (Table 1). Polymerase chain reaction was run to amplify each locus separately without using multiplex PCR. Female DNA and water were used as negative controls. PCR was carried out in a total volume of 20 μl and the reaction mixture included 40 ng of each DNA sample, 1 × PCR buffer, 1.5 mmol/l MgCl2, 200 μmol/l dNTP 1 μmol/l of each primer pair and 1 U Taq DNA polymerase (InVitrogene, USA). The reactions were carried out in a thermal cycler (MJ Research, USA). After an initial denaturation step at 94°C for 5 min, PCR amplification of 35 cycles were performed as follows: 95°C for 1 min for denaturation, 55–62°C ramping for 80 sec for primer annealing and 72°C for 60 sec for extension. The programs were followed by the final extension step at 72°C for 7 min. The reaction products were then analyzed by electrophoresis at 100 V on 2% agarose gels (Sigma, USA). In case a deletion was observed, a second run was performed in order to confirm the deletion in the presence of a positive control for that deletion.

**Table 1:** Cytogenetic abnormalities observed in infertile men with their semen analysis and Y chromosome microdeletion status. (NA-not applicable; NP-not performed)

Patients	064	0118	0139	0180	0211	0219	0185	0156	0295	0199
Karyotyping	46,XY,inv(1) (q12 or q21q42)	46,X,inv(Y) (p11.3q11.2)	47,XYY	47,XXY	47,XXY	47,XXY	45,X/46,XX mosaicism	46,X, t(X;Y) (p22.3;p11.2)	45,XY, der(13;14) (q10;q10)	46,XY, t(1,12) (q25;q24.3)
Y chromosome microdeletion	None	None	None	None	None	None	SY160 in AZFc	All deleted	None	None
Semen analysis	Severe oligozoospermia	Azoospermia	Severe oligozoospermia	Azoospermia	Azoospermia	Azoospermia	Severe oligozoospermia	Azoospermia	oligozoospermia	Severe oligozoospermia
Testicular biopsy (wet preparation)	NA	No sperm seen	NA	NP	Sperm found	No sperm seen	NA	NP	NA	NA
Testicular Volume: left right	12 12	15 15	15 20	3 3	6 6	12 12	15 18	8 10	NP	12 12
FSH (IU/L)	3	14	12	36	21	15	8	23	NP	6

## Results

A total of 257 patients were studied. Of those, 113 were azoospermic men. Among 144 oligozoospermic men, 110 were severe oligozoospermic (<1 × 10^6^/ml) and remaining 34 had a sperm count between 1 × 10^6 ^and 10 × 10^6^/ml. Ten (3.9%) patients had chromosome rearrangements. The results of cytogenetic analysis in 10 infertile men are shown in Table 1. Three cases showed 47,XXY karyotype consistent with Klinefelter syndrome, one case with 47,XYY, one case with 45,X/46,XX mosaicism, two cases with pericentric inversions of chromosome 1 and Y, one case of t(X;Y) involving the pseudo-autosomal region and the SRY gene confirmed by molecular cytogenetic techniques and two cases of apparently balanced reciprocal translocation involving autosomes. The two patients who had 45,X/46,XX mosaicism and t(X;Y) also showed Y chromosome microdeletions by PCR. Semen analysis in the whole group with chromosome rearrangements ranged from azoospermia to oligozoospermia. Three out of 5 azoospermic men had testicular biopsy performed for the identification of sperm in wet preparation, only one was positive for the presence of sperm (Table 1).

**Figure 1:**
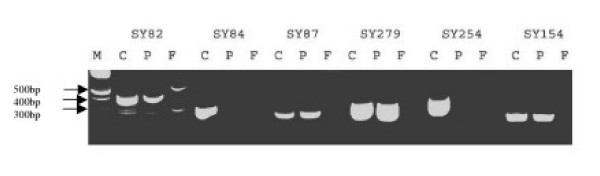
a gel electrophoresis showing coexistence of AZF a and c point deletions in the patient 8. in the previous and the following STSs of deleted region were run together with the deleted STSs. M: marker; C: positive control (normal male); F: female DNA. Note the absence of any amplifications in SY84 and SY254. They are similar to female profile.

Eight patients (3.2%) out of remaining 247 with normal karyotypes and no known causes of impaired spermatogenesis had Y chromosome microdeletions. Six patients had deletions in AZFc region, one had a deletion in AZFb and the other had both AZFa and AZFc deletions (Table 2). There were large deletions in six patients covering many STSs, among these, five were almost similar covering most of the AZFc region and one showed complete absence of AZFb markers used in this study. Regarding the small deletions, one STS in AZFa and one in AZFc region were missing in a patient (Fig 1) while in another, only one AZFc STS marker was deleted (Table 2). Fifty-three DNA samples extracted from patients with normal sperm count, motility and morphology showed absence of any deletions using the same STS markers.

**Table 2:** Schematic overview of the Yq11 microdeletions detected in 8 patients with unexplained oligo- or azoospermia. Positions of sequence-tagged sites (STS) and their location in AZF are also shown. Negative signs (-) indicate deletion of a STS; positive signs (+) indicate presence of a marker. The results of semen analysis and wet preparation testicular biopsy sperm search are shown in the last two columns (NA-not applicable; NP-not performed).

	Markers	AZF	1	2	3	4	5	6	7	8
1	SY82	**A**	+	+	+	+	+	+	+	+
2	SY84		+	+	+	+	+	+	+	-
3	SY87		+	+	+	+	+	+	+	+
4	SY125	**B**	+	+	+	+	+	+	-	+
5	SY129		+	+	+	+	+	+	-	+
6	SY132		+	+	+	+	+	+	-	+
7	SY134		+	+	+	+	+	+	-	+
8	SY136		+	+	+	+	+	+	-	+
9	SY143		+	+	+	+	+	+	-	+
10	SY130		+	+	+	+	+	+	-	+
11	SY153	**C**	+	-	-	-	-	-	+	+
12	SY148		+	-	-	-	-	-	+	+
13	SY277		+	-	-	-	-	-	+	+
14	SY279		+	-	-	-	-	-	+	+
15	SY254		+	-	-	-	-	-	+	-
16	SY154		+	-	-	-	-	-	+	+
17	SY157		-	-	-	-	-	-	+	+
18	SY158		+	-	-	-	+	+	+	+
19	SY160		+	+	+	+	+	+	+	+
Semen analysis			Severe oligozoospermia	Severe oligozoospermia	Azoospermia	Azoospermia	Severe oligozoospermia	Severe oligozoospermia	Azoospermia	Severe oligozoospermia
Testicular biopsy			NA	NA	No sperm	No sperm	NA	NA	NP	NA

## Discussion

Y chromosome microdeletions have been in the increasing interests of clinicians and scientists after ICSI was introduced to be the main treatment option for severe male factor infertility. Earlier studies showed high incidence of Y chromosome microdeletions in idiopathic oligo- or azoospermia [11-13] which prompted many researchers to screen their populations. The frequency of deletions was reported to be in the range of 0.7–34.5 % with an average of 8.2% [6]. The results shown in this study (8 out of 247; 3.2%) is in the lower part of this range. The wide range of the deletions in different publications was suggested to be due to the ethnic composition of the study populations in various studies. [14,15]. In addition, the inclusion criteria could have also played an important role in this variable frequency of deletion. Furthermore, different protocols and the number of markers used in the studies might have further contributed to the variation. Karyotyping was also performed and the prevalence of abnormality was estimated to be 3.96% (10/257). This is slightly lower than other reports [16,17].

Of the 247 patients with normal karyptypes and no known cause of infertility, 3/108 (2.8%) azoospermic and 5/139 (3.6%) of oligozoospermic had Y chromosome microdeletions, respectively. Although the rate of deletion was similar in these two groups in our study, Y chromosome microdeletions in azoospermic patients was found to be approximately two-fold more than oligozoospermic ones in earlier studies [6]. This discrepancy again could be due to our patient population or the threshold of sperm concentration included for Y chromosome microdeletion analysis. Our inclusion criteria were less stringent since all idiopathic cases with less than 10 × 10^6^/ml sperm count were included in the study. All the deletions observed were in the severe oligozoospermic patients (<1 × 10^6^/ml, 5/106, 4.7%) while none of 33 patients with sperm count between 1 and 10 × 10^6^/ml had any deletions. Similarly, none of the 53 normozoospermic men included as normal control had any deletions in our population. This indicates that none of the observed deletions in our study is due to polymorphisms. Furthermore, overall deletion in fertile controls has been reported to be less than 0.5% in previously published reports [6,18].

Five out of 8 patients with Y chromosome microdeletions have almost the entire AZFc region deleted. The infertility phenotype of patients with large AZFc deletions varied from azoospermia (2 patients) to severe oligozoospermia (3 patients). It has been shown that reduction of the sperm number leading to azoospermia might be secondary to time dependent germ cell regression [3,19]. In this report, however, we did not see any trend of phenotype changes according to ages.

One of the patient was carrying a deletion of the entire AZFb. This deletion is larger than the one published by Ferlin et al. [20]. The patient with AZFb deletion showed an azoospermic phenotype which is in accordance with the suggestions stating that AZFb deletion has an adverse prognosis for finding sperm in testicular biopsies [21,22].

One patient with severe oligozoospermia in this study had deletions of 2 STSs (one AZFa and other AZFc). These deletions are unique and to the best of our knowledge this is the first report showing AZFa and AZFc point deletions in the same individual. Repeated testing with different controls consistently confirmed the deletions in this patient. Although deletions occurring in AZFa are mostly associated with Sertoli cell only syndrome [23], severe oligozoospermia in our patient with AZFa and AZFc deletions was not surprising because Kamp et al. [23] also reported that only complete AZFa deletion is associated with the absence of sperm whereas there have been cases of sperm retrieval with partial AZFa deletions.

Most deletions are de novo in origin and the transmission of these deletions to the male offspring is 100% [24-27]. The percentage of Y chromosome microdeletions is lower in our population; however, the common use of ICSI for severe male factor infertility necessitates a proper counseling.

In conclusion, Y chromosome microdeletions and chromosomal rearrengements are low in our population, contributing to a small percent of idiopathic oligo- and azoospermia. This warrants that other additional genetic, epigenetic, nutritional and local factors to be investigated in the etiology of idiopathic oligo- or azoospermia.
